# The loss of neoantigens is an important reason for immune escape in multiple myeloma patients with high intratumor heterogeneity

**DOI:** 10.1002/cam4.6721

**Published:** 2023-11-15

**Authors:** Yue Wang, Jiadai Xu, Tianwei Lan, Chi Zhou, Peng Liu

**Affiliations:** ^1^ Department of Hematology, Zhongshan Hospital Fudan University Shanghai China

**Keywords:** immune escape, intratumor heterogeneity, multiple myeloma, neoantigen, tumor mutation burden

## Abstract

**Objectives:**

Intratumor heterogeneity (ITH) is an important factor for clinical outcomes in patients with multiple myeloma (MM). High ITH has been proven to be a key reason for tumor immune escape and treatment resistance. Neoantigens are thought to be associated with ITH, but the specific correlation and functional basis for this remains unclear.

**Methods:**

We study this question through the whole‐exome sequencing (WES) data from 43 high ITH newly diagnosed MM patients in our center. Mutant allele tumor heterogeneity (MATH) was conducted to quantify ITH. The cutoff value for high intratumor heterogeneity was determined by comparing MATH of different kinds of tumors. NeoPredPipe was performed to predict neoantigens and binding affinity.

**Results:**

Compared to other tumors, MM has a relatively low tumor mutation burden but a high ITH. Patients with high MATH had significantly shorter progression‐free survival times than those with low MATH (*p* = 0.001). In high ITH samples, there is a decrease in strong‐binding neoantigens (*p* = 0.019). The loss of strong‐binding neoantigens is a key factor for insensitivity to therapy (*p* = 0.015). Loss of heterozygosity in HLA was not observed. In addition, patients with fewer neoantigens loss had higher rates of disease remission (*p* = 0.047). CD8 + T cells (*p* = 0.012) and NK cells (*p* = 0.011) decreased significantly in patients with high neoantigens loss rate. A prediction model based on neoantigens was built to evaluate the strength of immune escape.

**Conclusion:**

The loss of strong‐binding neoantigens explains why tumors with high ITH have a higher degree of immune escape and may be feasible for deciding the clinical treatment of MM.

## 1 INTRODUCTION

Intratumor heterogeneity (ITH) is the result of constant mutations in tumor development, resulting in internal biological behavior and gene diversity.^1^, ^2^ High ITH is considered to be a marker of poor prognosis and is closely associated with drug resistance and immune escape in patients.^1^, ^3^, ^4^, ^5^ Immunotherapy plays an important role in antitumor therapy. The application of immunomodulators, chimeric antigen receptor T‐cell immunotherapy (CART), and immune checkpoint inhibitors have brought clinical benefits to many patients. The essence of immunotherapy is the specific recognition and killing effect of effector T cells on tumor cells.^6^ The activation of immune response relies on the production of neoantigen, antigen‐presenting, and neoantigen recognized by T cells.^7^, ^8^, ^9^ Previous studies have confirmed that neoantigens are one of the important factors in immune response and immunotherapy.^7^, ^10^, ^11^ Hypothesis in the immunotherapy field holds the opinion that tumors with increased tumor mutation present more neoantigens and are more immunogenic.^8^ However, tumors containing equal mutation burden levels vary greatly in the number of neoantigens and responses to treatment.^12^, ^13^ In summary, the strength of the antitumor immune response does not depend simply on the number of mutations but on the complex subsequent effects of T cells on the mutations.^14^, ^15^ Neoantigen production does not depend solely on mutational coincidence. The structure of neoantigens and their subsequent immune responses are highly diverse.^16^, ^17^ According to the type of mutation, the mutation of a single nucleotide site may produce a specific peptide chain with immune effect potential, and the mutation of different sites produces a variety of neoantigens, and at the same time produces a variety of binding abilities to T cells. This part of neoantigens is the part that has been studied more. The binding ability of neoantigens to T cells is one of the important factors for the effective immune response produced by neoantigens.^18^ By contrast, insertion and deletion mutations can produce a large number of continuous protein‐coding changes.^8^ The immune response of the two different mutation modes is different, but it still lacks in‐depth exploration. In addition, the dysfunction of antigen presentation caused by the loss of heterozygosity of HLA, as well as the activation, dysfunction, and exhaustion of T cells under the long‐term antigen stimulation of tumor also play a key role in the body's antitumor immunity.^19^, ^20^ The diversity of neoantigens theoretically reflects the heterogeneity within tumors. High ITH tumors are often accompanied by strong immune escape.^21^, ^22^ However, it is still unclear how ITH affects the diversity of neoantigens.

Multiple myeloma (MM) is a malignant tumor that originates from B cells.^23^ Compared to a solid tumor, like lung cancer or breast cancer, the incidence of MM is extremely low, which is 1.03 per 100,000 in China.^24^ However, MM is the second most common malignant tumor in the blood system, and the research progress is relatively backward. As a tumor of the immune system, immune responses, and neoantigens play a more important role in myeloma than in other solid tumors.^25^ With the development of immunomodulatory drugs, the prognosis of MM patients improved a lot. However, the prognosis of patients with high ITH is still very poor.^26^ And the response to treatment in patients with high ITH differs substantially from those with low ITH.^27^ The characteristics of tumor immune escape, the characteristics of the body's immune status, and the characteristics and causes of immune escape in MM patients with high ITH remain to be explored. Therefore, it is of great significance to explore the high ITH of MM patients. Studies' effort to combine the ITH and neoantigens is completely blank in MM.

In this study, we quantified ITH in newly diagnosed MM patients by whole‐exome sequencing and screened out MM patients with high ITH by multitumor species comparison. We explored the neoantigens produced by mutation in MM patients with high ITH, explored the occurrence of the loss of heterozygosity in human leukocyte antigen (HLA‐LOH), and compared the immune status of the body under different neoantigen levels. We investigated the relationship between ITH and neoantigens and its effect on immune response and immune escape. We also investigated the effects of neoantigens on the treatment and prognosis of patients with high ITH and attempted to establish a system for determining the prognosis of patients based on intratumor heterogeneity and neoantigen escape index.

## 2 MATERIALS AND METHODS

### 2.1 Patient enrollment

High‐heterogeneity tumors are characterized by poor prognosis and severe clinical organ damage, and patients with high tumor burden are more likely to have complex ITH. Since we could not determine the level of ITH before high‐throughput sequencing, and the Durie‐Salmon (DS) staging system is based on the tumor burden of myeloma,^28^ we speculated that patients with DS stage III are more likely to have high ITH. We collected specimens from patients with DS stage III and underwent immunomagnetic bead sorting of bone marrow specimens and whole‐exome sequencing to ultimately identify individuals with high ITH. To further explore the ITH and neoantigens evolution during the development and progression of myeloma, we also included samples of its precursor disease status and recurrence and progression status. This study included 49 newly diagnosed DS stage III MM patients who received standard first‐line therapy and did not undergo autologous stem cell transplantation, seven patients with relapsed or refractory MM, and seven patients with newly diagnosed monoclonal gammopathy of unknown significance between July 2017 and October 2019 at the department of hematology, Zhongshan hospital Fudan University. A total of 63 patient specimens were included in the study. The patients with relapsed myeloma all developed disease progression after receiving first‐line treatment. All patients did not diagnose with other malignant tumors and patients with amyloidosis were also excluded. All enrolled patients were voluntarily admitted to our hospital for regular follow‐up and had an ECOG score of 0–2 and a life expectancy of more than 3 months. Newly diagnosed patients were treated with a three‐drug combination of first‐line regimens containing proteasome inhibitors, immunomodulators, and dexamethasone. Patients underwent bone marrow biopsy and puncture before inclusion in the study, and bone marrow specimens were taken for subsequent studies. The study was approved by the Ethics Committee of Fudan University Zhong Hospital and complied with the principles of the Helsinki Accord. This was a retrospective study and patient consent was therefore deemed unnecessary. However, bone marrow samples were collected from all subjects, with patient consent, and stored in the tissue bank at Fudan University Zhongshan Hospital.

In the validation set, clinical data and mutation gene data were acquired from MM Research Consortium (MMRC) institutions.^26^ Sequencing data were acquired from the dbGaP database (http://www.ncbi.nlm.nih.gov/gap) under accession number phs000348. Two hundred and one patients were enrolled in the validation set. Two cases in the dataset were excluded due to incomplete information on mutation genes, which made it impossible to calculate the MATH score.

### 2.2 Bone marrow specimen and somatic mutation detection

Bone marrow from all enrolled patients underwent CD138 magnetic bead sorting to ensure that the proportion of monoclonal plasma cells was greater than 70%. CD138‐positive cells after sorting were stored in the cell preservation solution and underwent whole‐exome sequencing. Peripheral blood cells from the patients were collected and used as control specimens for somatic mutation detection. FASTQ files were analyzed based on the GRCh37 reference genome. Whole‐exome sequencing was aligned using the mem module of Burrows‐Wheeler Alignment Tool (BWA) version 0.7.9.^29^ Alignment files were based on quality score recalibrated and locally realigned around indels with the Genome Analysis Toolkit (GATK) version 4.2.0^30^ and MuTect version 2.1.^31^ Consensus genotype calls were generated using SAMtools version 1.11^32^ and annotated using the Annovar package.^33^ Bcftools version 1.5 was used for variant calling and manipulating VCFs.^34^ Somatic events identified in 63 patients with MM and other evolution stages of plasmacytoma by whole‐exome sequencing are shown in Data S1.

### 2.3 Intratumor heterogeneity and mutant allele tumor heterogeneity score

Because MM is not a solid tumor, ITH cannot be assessed by multipoint sampling, we used the mutant allele tumor heterogeneity (MATH) score to quantify the intratumoral heterogeneity of patients.^35^ MATH score is a measurement of ITH, which calculates the width of the Variant Allele Frequency (VAF) distribution by whole‐exome sequencing of a single sample. It is calculated as the percentage ratio of median absolute deviation (MAD) and the median of its mutant allele fractions at tumor‐specific mutated loci. It is based on the data of the tumor and matched normal DNA: MATH = 100*MAD/median. And the calculation of MAD followed the default in R, with values scaled by a constant factor (1.4826) so that the expected MAD of a sample from a normal distribution equals the standard deviation. Here we used maftools packages to figure up the MATH score which contained a clustering algorithm to enhance the accuracy of the genomic pattern.^36^

### 2.4 Neoantigen prediction, binding affinity, immune response, and HLA‐LOH

Patients' HLA class I and class II predictions were using HLAminer, a pipeline for predicting HLA from shotgun sequence data.^37^ HLA‐A, ‐B, and ‐C haplotypes were identified for further analysis. The patient's HLA‐LOH was progressively calculated using the LOHHLA package.^20^ Corresponding wild‐type peptide sequences using netMHCpan‐4.1.^38^ It is a server that predicts the binding of peptides to any MHC molecule of the known sequence using artificial neural networks (ANNs). Patient‐specific neoantigen was determined separately based on mutation detection and HLA haplotypes from exome sequencing. All types of somatic mutations in genes with protein products longer or equal to 8 amino acids were used to produce peptide sequences of 8, 9, and 10 amino acids, which contain mutations by tiling across the peptide sequence. The steps outlined above deliver candidate information for neoantigens which might be presented to cytotoxic T‐cells. However, the ability of tumor neoantigens to bind to T cells predicted by the above procedure cannot be well determined. The potential neoantigen results were further integrated through software called NeoPredPipe,^39^ a contiguous means of predicting putative neoantigens and their corresponding recognition potentials for both single and multiregion tumor samples. This method defined the recognition potential as the product of A and R. A is the amplitude of the ratio of the relative probabilities of binding for the wild‐type and mutant epitopes to the MHC class I molecules; R is a measure of similarity to pathogenic peptides which represent the probability that the neoantigen in question is recognized by a TCR clone already present in the tissue. Finally, the tumor neoantigens which might induce an immune response were identified, and the neoantigens were divided into the strong‐binding and weak‐binding according to recognition potential. The peptide will be identified as a strong binder if the % Rank is below the specified threshold for the strong binders, by default 0.5%. The peptide will be identified as a weak binder if the % Rank is above the threshold of the strong binders but below the specified threshold for the weak binders, by default 2%. Based on different mutation types, the neoantigens generated by single nucleotide mutations and indels will be displayed and explored separately.

### 2.5 Identification of peripheral blood lymphocyte subsets, cytokines, and complement of patients

The peripheral blood lymphocyte subsets were determined through flow cytometry from a peripheral blood sample obtained from the patient at diagnosis (BD MultiTEST IMK Kit). CD19(+) B lymphocytes, CD3(+) T lymphocytes, CD4(+) T lymphocytes, CD8(+) T lymphocytes, and CD56(+)CD16(+) NK cells were identified with the use of fluorescent staining for lymphocyte surface molecules. The levels of cytokines, complement C3, and complement C4 in the peripheral blood of patients were detected by ELISA (Invitrogen ELISA kit). The cytokines detected included IL‐1β, sIL‐2R, IL‐6, IL‐8, and IL‐10.

### 2.6 Statistical analysis

Statistical methods were all analyzed by R version 4.0.3 (http://cran.r‐project.org) and Stata statistical software, version 14.0 (StataCorp, College Station, TX). *p* values were all two‐sided and statistical significance was set at *p* < 0.05 if not mentioned. All confidence intervals (CIs) were stated at the 95% confidence level.

Log‐rank test and Kaplan–Meier survival estimates were applied to compare the progression‐free survival between different groups. Kendall and Spearman's analysis was used to analyze the correlation between the two parameters. Multivariate Cox regression was performed to analyze the predicted value of the MATH score or neoantigens for patients' prognoses. Student *t*‐test or Wilcoxon rank‐sum test was used to determine the difference of parameters between two different groups according to whether they fit the normal distribution. Due to the unavailability of individual survival and progression times in the validation set, only relevant data on disease progression in patients can be obtained, the least absolute shrinkage and selection operator analysis (LASSO) was applied to identify whether MATH could predict patients' prognosis.

## 3 RESULTS

### 3.1 Clinical characteristics of patients

In the training set, the median age of the 63 patients was 64, ranging from 41 to 87 years old. The median follow‐up time of this cohort was 16.7 months, ranging from 4.1 to 37.3 months. All 49 newly diagnosed myeloma patients received a combination regimen of proteasome inhibitors, immunomodulators, and dexamethasone. 18 (36.7%) patients staged as ISS stage I, 11 (22.4%) patients staged as ISS stage II, and 20 (40.8%) patients staged as ISS stage III. A total of 28 (57.1%) were male and 21 (42.9%) were female. Nine patients died and 23 patients progressed during follow‐up. Seven patients with monoclonal gammopathy of unknown significance (MGUS) did not meet the diagnostic criteria for myeloma and therefore were not treated and received regular intensive follow‐ups. All seven relapsed and refractory multiple myeloma (RRMM) patients were previously diagnosed with MM and were treated with a three‐drug combination first‐line regimen containing a proteasome inhibitor that was judged to be disease progression before receiving second‐line therapy. The clinical characteristics of patients are shown in Table 1.

**TABLE 1:** Baseline characteristics of patients in the training cohort.

Variable	Total	MM	HIMM	MGUS	RRMM
Patients (No)	63	49	43	7	7
Gender (No, %)					
Male	37 (58.7)	28 (57.1)	22 (51.2)	5 (71.4)	4 (57.1)
Female	26 (41.3)	21 (42.9)	21 (48.8)	2 (28.6)	3 (42.9)
Age(No, %)					
<60	24 (38.1)	20 (40.8)	17 (39.6)	2 (28.6)	2 (28.6)
≥60	39 (61.9)	29 (59.2)	26 (60.4)	5 (71.4)	5 (71.4)
ISS stage(No, %)					
I	26 (41.3)	18 (36.7)	17 (39.5)	7 (100.0)	1 (14.3)
II	13 (20.6)	11 (22.4)	10 (23.3)	0 (0.0)	2 (28.6)
III	24 (38.1)	20 (40.8)	16 (37.2)	0 (0.0)	4 (57.1)
Heavy chain(No, %)					
IgG	39 (61.9)	28 (57.1)	24 (55.8)	6 (85.7)	5 (71.4)
IgA	18 (28.6)	15 (30.6)	14 (28.6)	1 (14.3)	2 (28.6)
IgD	2 (3.2)	2 (4.1)	1 (2.3)	0 (0.0)	0 (0.0)
FLC	4 (6.3)	4 (8.2)	4 (9.3)	0 (0.0)	0 (0.0)
Light chain (No, %)					
Kappa	19 (30.1)	16 (32.7)	14 (32.6)	0 (0.0)	3 (42.9)
Lamda	34 (54.0)	28 (57.1)	24 (55.8)	2 (28.6)	4 (57.1)
None	10 (15.9)	5 (10.2)	5 (11.6)	5 (71.4)	0 (0.0)
FISH high‐risk (No, %)					
Detected	14 (22.2)	11 (22.4)	9 (20.9)	0 (0.0)	3 (42.9)
Not detected	49 (77.8)	38 (77.6)	34 (79.1)	7 (100.0)	4 (57.1)
Follow‐up time (median, range)	16.7 (3.5–37.3)	16.7 (4.1–37.3)	16.7 (4.1–37.3)	16.2 (11.2–20.0)	13.5 (3.5–19.9)
Progress (No, %)	29 (46.0)	23 (46.9)	20 (46.5)	0 (0.0)	6 (85.7)
Dead (No, %)	12 (19.0)	9 (18.4)	8 (18.6)	0 (0.0)	3 (42.9)

In the validation set, a total of 201 patients were enrolled in the study. The median age of these patients was 61, ranging from 24 to 82 years old. Among the enrolled patients. A total of 128 (63.7%) were male, 67 (33.3%) were female, and 6 (3.0%) were not available. Forty‐one patients died and 35 patients progressed during follow‐up. The clinical characteristics of patients are shown in Table S1.

### 3.2 Somatic mutation gene comparison and validation

To verify the accuracy and representativity of the next‐generation sequencing data of this study, we compared the sequencing data from this study with the data from large‐scale myeloma samples in other centers. In the training set, the median variants per sample were 42, and the most common mode of single nucleotide variations in C to T, which was similar to the data in the validation set (Figures S1 and S2). Genes with a mutation rate of more than 5% overlapped highly in the two sets. These results indicated that our sequencing data queue has good credibility and representativeness. The mutational characteristics of 49 newly diagnosed myeloma patients were summarized in Figure S1.

### 3.3 Mutant allele tumor heterogeneity score and the definition of high intratumoral heterogeneity

**FIGURE 1:**
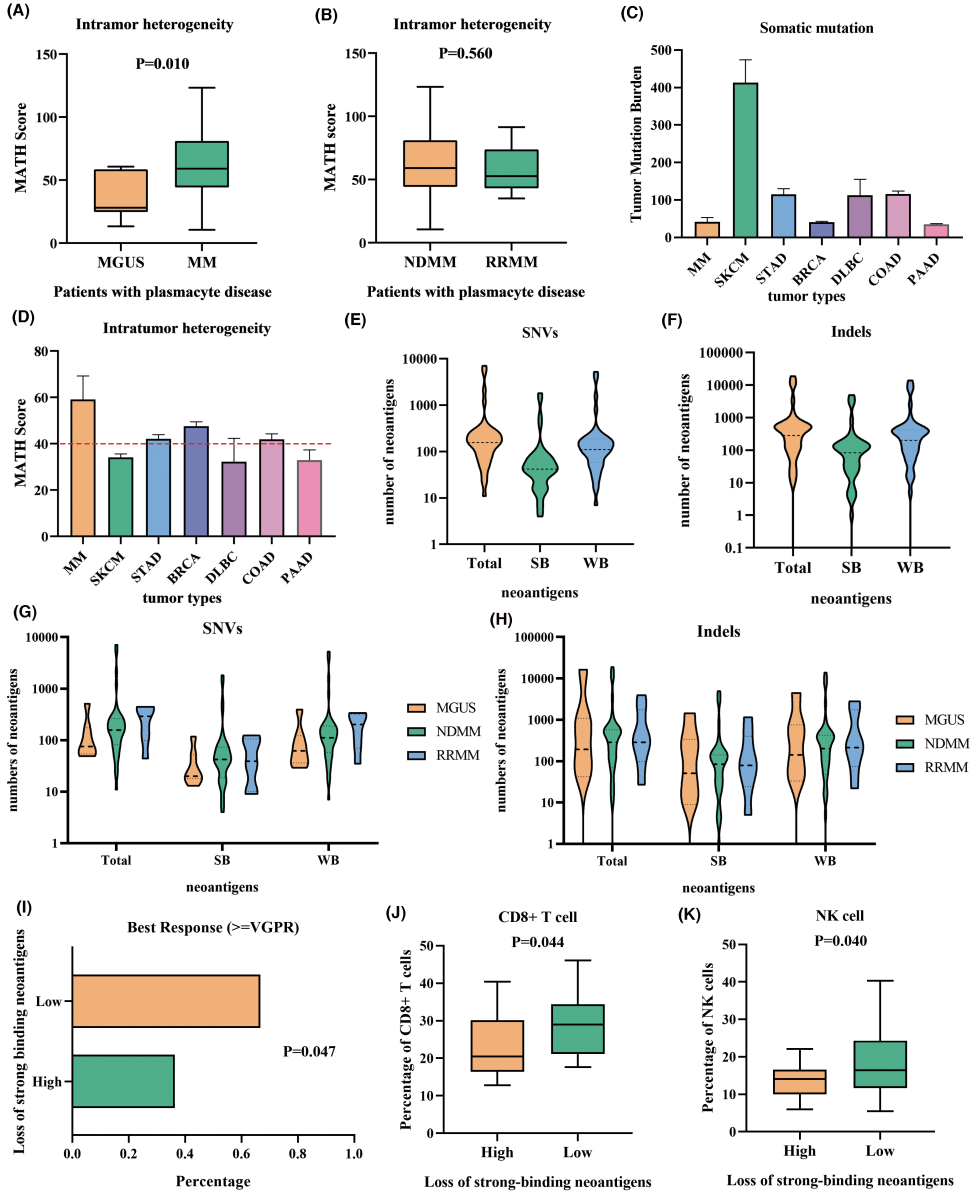
MATH score, tumor mutation burden and neoantigens in myeloma. (A) MATH score in patients with MGUS and newly diagnosed MM. (B) MATH score in patients with newly diagnosed MM and recurrence MM. (C) Tumor mutation burden in different type of tumor. (D) MATH score in different type of tumor. (E) The number of neoantigens produced by SNVs in MM patients with high ITH. (F) The number of neoantigens produced by indels in MM patients with high ITH. (G) The number of neoantigens produced by SNVs in MGUS, NDMM, and RRMM patients. (H) The number of neoantigens produced by indels in MGUS, NDMM, and RRMM patients. (I) The best outcome achieved during the first‐line treatment in different strong‐binding neoantigens group. (J) The proportion of CD8+ T cells in different neoantigen deletion groups. (K) The proportion of NK cells in different neoantigen deletion groups. BRCA, breast invasive carcinoma; COAD, colon adenocarcinoma; DLBC, diffuse large B‐cell lymphoma; MATH, mutant allele tumor heterogeneity; MGUS, monoclonal gammopathy of unknown significance; MM, multiple myeloma; NDMM, newly diagnosed multiple myeloma; PAAD, pancreatic adenocarcinoma; RRMM, relapsed and refractory multiple myeloma; SKCM, skin cutaneous melanoma; STAD, stomach adenocarcinoma.

In the training cohort, the median MATH value of 49 newly diagnosed myeloma patients was 59.1, ranging from 10.54 to 123.3. The median MATH value of seven newly diagnosed MGUS patients was 28.0, ranging from 13.27 to 60.7. Wilcoxon rank‐sum test showed that there was a significant difference in MATH between MGUS and MM patients (*p* = 0.010). The intratumoral heterogeneity of MM patients was higher than that of MGUS patients (Figure 1A), but there was no significant difference in tumor mutation burden (TMB) between the two groups. The median value of MATH in seven patients with RRMM was 52.65, ranging from 35.08 to 91.48, and there was no significant difference in MATH between newly diagnosed myeloma and RRMM (Figure 1B). And in the validation cohort, the median MATH value of 201 myeloma patients was 52.0, ranging from 9.5 to 138.1. Due to the disunity of inclusion standards and treatment of patient specimens in the validation cluster, we did not perform further subgroup comparisons. We found that the median and mean MATH values of patients in our center were significantly higher than those in the MMRC set, demonstrating the success of our strategy to screen for high ITH myeloma based on high tumor burden.

**FIGURE 2:**
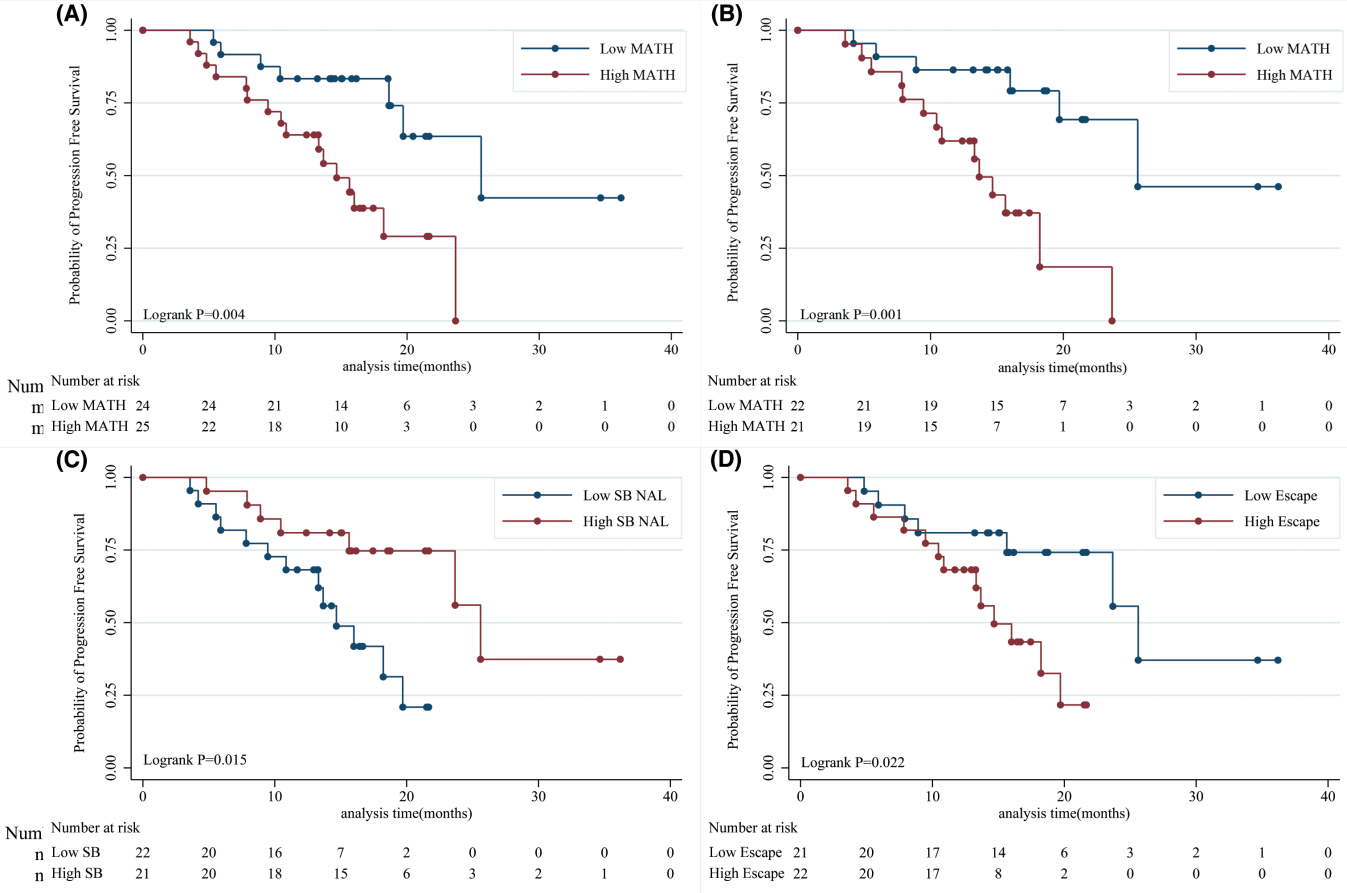
Kaplan–Meier plots of PFS in different group. (A) Kaplan–Meier plots of PFS in 49 newly diagnosed myeloma patients received first‐line therapy. (B) Kaplan–Meier plots of PFS in in 43 newly diagnosed myeloma patients with high ITH. (C) Kaplan–Meier plots of PFS in high ITH patients with different number of strong‐binding neoantigens. (D) Kaplan–Meier plots of PFS in newly diagnosed myeloma according to different immune escape group. ITH, intratumor heterogeneity; PFS, progression‐free survival.

To test whether the MATH score could well predict the prognosis and well quantify the ITH of patients, we divided the patients into low and high heterogeneity groups according to the median value of MATH value in 49 newly diagnosed MM patients. Survival curves were constructed using the Kaplan–Meier method, with log‐rank tests used to assess the differences between the groups. As a result, of all newly diagnosed myeloma patients, a higher MATH score (*p* = 0.004) was associated with a higher possibility of disease progression (Figure 2A). Multivariate Cox analysis which included MATH score, age, gender, ECOG score, cytogenetic abnormalities, ISS stage, hyperdiploid, and type of light chain also found that the MATH score was an independent predictor of PFS (*p* = 0.037) (Table 2). These results indicated that the MATH score can well quantify ITH in patients with myeloma.

**TABLE 2:** Multivariate Cox proportional hazards analysis of progression‐free survival time for Patients in the training set.

Variables	PFS		
	HR	95% CI	*p*
MATH	1.022	1.001–1.043	0.037
FISH high‐risk	0.962	0.282–3.277	0.950
ISS stage	2.117	0.996–4.498	0.051
Gender	0.942	0.319–2.774	0.913
Age	0.971	0.920–1.024	0.276
ECOG	1.837	0.627–5.381	0.267
Hyperdiploid	2.528	0.749–8.530	0.135
Lightchain	0.984	0.419–2.307	0.970

In the validation set, the information on individual survival and progression time was unavailable, and only categorical variable data on whether patients have developed disease progression can be obtained. Lasso analysis which contained age, light chain type, hyperdiploid, MATH score, prior MGUS, previous treatment, and gender was performed to explore whether the value of MATH was associated with disease progression. The endpoint was progression‐related death. The result indicated that the MATH score was an independent predictor of PFS. Logistic regression which included the same parameters as Lasso analysis was also carried out to further verify our results. The result indicated that only the MATH score (*p* = 0.015) and previous treatment (*p* < 0.0001) were related to patients' PFS (Figure S3).

We performed a more refined subgroup analysis of 201 patients in the MMRC database for whom MATH values could be calculated. Of these 201 patients, only three were diagnosed with MGUS. Specific treatment information and sample sampling times for all MM patients in the MMRC cohort are unknown, and only information on whether they were receiving treatment at the time of sampling is provided. We were unable to determine whether these treated patients were sampled after relapse and progression, and therefore were unable to compare the MATH values of NDMM and RRMM patients. Therefore, we were only able to compare MATH between MGUS and MM patients. Due to the extremely small sample size of MGUS patients, only three individuals, which is not sufficient to meet the statistical power requirements, we only present the results for presentation purposes. The median MATH score of MGUS group was 40.3 (36.6–61.0), and the mean MATH score of MM group was 53.3 (9.5–138.1). The *p* value of the Wilcoxon rank‐sum test is 0.475. The MATH values of the MM group seem to be higher than those of the MGUS group, but it is difficult to draw firm statistical conclusions.

To define high ITH myeloma and explore the characteristics of MM at the level of gene mutation, we compared the MATH scores of MM to other tumor species in the TCGA database, including diffuse large B‐cell lymphoma (DLBCL), a type of malignant neoplasm also originating from B cells; Skin cutaneous melanoma (SKCM), the tumor with the highest mutation burden; Stomach adenocarcinoma (STAD), colorectal cancer (COAD) and pancreatic cancer (PAAD), tumor with high mutation conformance, high genetic heterogeneity and large sample size, and breast cancer (BRCA), the tumor with the largest sample size in TCGA (Figure 1C,D). By comparing the mutation profile with other tumors, we found that MM has a high level of intratumoral heterogeneity score despite having only a midrange mutation profile compared to other tumors (Figure S4). By comparing the interspecific ITH of multiple tumors in the TCGA database, we found that the MATH value of 40 was the median for the interspecific comprehensive analysis of multiple tumors. Finally, we defined the high intratumoral heterogeneity of MM with a MATH score greater than 40.0 (Figure 1D). Of the 49 newly diagnosed myeloma patients in our collection, 43 were defined as high ITH MM.

**FIGURE 3:**
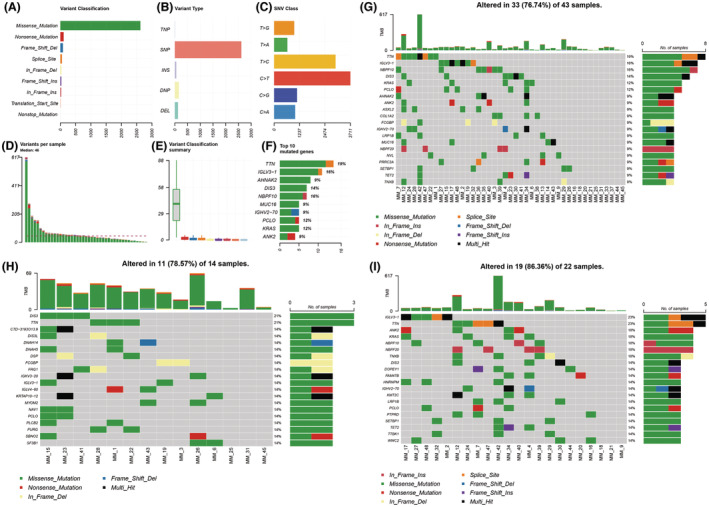
Baseline gene mutation characteristics of patients in high ITH MM patients. (A) The summary variant classification in 43 high ITH newly diagnosed myeloma patients. (B) The summary variant type in 43 high ITH patients. (C) The summary SNV class in 43 high ITH patients. (D) The summary tumor mutation burden in 43 high ITH patients. (E) The mutation rates of different variant classification in 43 high ITH patients. (F) Top 10 mutated genes and mutation rate in high ITH MM patients. (G) The top 20 mutated genes and mutation patterns in patients with high ITH. (H) The top 20 genes and mutation patterns in the poor prognosis group. (I) The top 20 genes and mutation patterns in the better prognosis group. ITH, intratumor heterogeneity; MATH, mutant allele tumor heterogeneity; SNV, single nucleotide variation.

We also separately calculated the prognostic value of MATH in 43 patients with high ITH. We found that patients with higher MATH scores (*p* = 0.001) were less likely to respond to treatment even in high ITH myeloma (Figure 2B). The summary mutation profile of 43 myeloma patients with high ITH is shown in Figure 3A–F.

### 3.4 Neoantigens, HLA‐LOH, and disease characteristics of patients

Although HLA‐LOH was found in about 40% of patients with non‐small cell lung cancer in the previous literature, no HLA‐LOH phenomenon was found in the samples of patients with high ITH myeloma in this study after the examination. In terms of neoantigens, among the neoantigens produced by single nucleotide variants (SNVs), the median total number of neoantigens of 43 high ITH newly diagnosed myeloma patients was 157, ranging from 11 to 7089. And the median number of strong‐binding neoantigens was 42, ranging from 4 to 1831 (Figure 1E). Among the neoantigens produced by indels, the median total number of neoantigens was 285, ranging from 0 to 18,950. And the median number of strong‐binding neoantigens was 84, ranging from 0 to 4957 (Figure 1F). We also used the old version of netMHCpan4.0 to validate the results and found no significant difference. We further analyzed whether the number of neoantigens correlated with certain clinical parameters. The results showed that there was no significant difference in neoantigens between different stages, gender, or light chain type. And the cytogenetic abnormalities were not associated with the number of neoantigens.

We also compared the neoantigens from patients with MGUS, MM, and RRMM in terms of their total number, number of strong‐binding neoantigens, and number of weak‐binding neoantigens. ANOVA analysis was used to compare the number of neoantigens among the three groups. The results indicated that there were no significant differences in the number of neoantigens among the three groups (SNVs: *p* = 0.5853; Indels: *p* = 0.1597). The expression of neoantigens in different groups can be found in Figure 1G,H.

### 3.5 High intratumor heterogeneity is closely related to the decrease of strong‐binding neoantigens produced by SNVs

Then we explored whether neoantigens were associated with ITH. We found that when the heterogeneity in patients is relatively high, the number of strong‐binding neoantigens produced by SNVs tends to decrease. Neoantigens which are produced by gene mutations are recognized by T cells and lead to antitumor‐associated immune responses. We hypothesized that in high ITH tumors, with the increase of intratumor heterogeneity, the neoantigens produced by SNVs that can be recognized and tightly bound by T cells decrease, which weakened the antitumor immune response of the body and made it less sensitive to immunotherapy. It is an important mechanism of tumor immune escape. However, a similar association was not observed for neoantigens produced by indels.

To test our hypothesis, both Kendall and Spearman correlation analyses were used to determine whether there was an association between the MATH score and the number of strong bind clonal neoantigens produced by SNVs in patients with high ITH. Among these 43 patients, Kendell correlation analysis showed that the MATH score was correlated with the number of strong‐binding neoantigens (*p* = 0.019). Kendall τb correlation coefficient was −0.249, indicating that the higher the heterogeneity of the tumor, the more neoantigen loss on the tumor cell surface. Similarly, the Spearman correlation also showed that the MATH score was closely related to the number of strong‐binding neoantigens (*p* = 0.019). Meanwhile, the Spearman rank correlation coefficient was −0.356, which also confirmed our hypothesis. These results indicate that the MATH score is negatively correlated with the number of strong‐binding neoantigens in high ITH myeloma patients. In patients with relatively high ITH, the loss of strong‐binding neoantigens is a phenomenon that cannot be ignored.

However, no statistically significant conclusions were observed in the correlation analysis between ITH and neoantigens produced by indels. The neoantigens generated by SNVs and indels are significantly different in terms of the immune response to tumors. At present, the subsequent immune effects of group neoantigens produced by indels cannot be determined. The mechanisms of immune escape from population neoantigens generated by insertion and deletion mutations and the heterogeneity of response responses are also poorly understood. Since we observed no meaningful phenomena, in subsequent analyses, we delved only into the neoantigens produced by SNVs.

### 3.6 The loss of strong‐binding neoantigens was a prognostic factor for disease progression in high intratumor heterogeneity patients

In high ITH patients, to test whether the number of strong‐binding neoantigens produced by SNVs was associated with prognosis and treatment response, we divided the patients into low and high strong‐binding neoantigens groups according to the median number. We constructed Kaplan–Meier survival curves and log‐rank tests to assess the differences between the groups. As a result, fewer neoantigens (*p* = 0.015) were associated with a higher possibility of disease progression (Figure 2C). In addition, multivariate Cox analysis after adjustment for all the potential prognostic factors which included numbers of strong‐binding neoantigens, age, gender, ECOG score, cytogenetic abnormalities, ISS stage, hyperdiploid, and type of light chain was performed (Table 3). The result indicated that a reduction in the number of strong‐binding neoantigens was associated with poorer treatment response (*p* = 0.037).

**TABLE 3:** Multivariate Cox proportional hazards analysis of progression‐free survival time for patients with high ITH in the training set.

Variables	PFS		
	HR	95% CI	*p*
SB Neoantigen	0.982	0.966–0.999	0.037
FISH high‐risk	1.074	0.286–4.035	0.915
ISS stage	2.677	1.182–6.062	0.018
Gender	1.038	0.328–3.287	0.949
Age	1.008	0.959–1.060	0.745
ECOG	5.086	1.462–17.697	0.011
Hyperdiploid	1.592	0.330–7.679	0.562
Lightchain	0.714	0.315–1.615	0.418

Since all 43 patients were treated with the same regimen, we further evaluated the effect of the loss of strong‐binding neoantigens on the remission rate of the treatment. We divided patients into the good response group (greater than VGPR) and the poor response group (not achieving VGPR) according to the best outcome achieved during the first‐line treatment. The best response above VGPR was achieved in eight patients (36.4%) in the group with more neoantigen loss and 14 patients (66.7%) in the group with fewer neoantigens loss (Figure 1I). By chi‐square test, we found that patients with fewer neoantigens loss had higher remission rates (*p* = 0.047).

### 3.7 Relationship between neoantigens and mutated genes

We further explored the relationship between the number of neoantigens and the mutated genes. In our analysis, we found that the total number of neoantigens (*p* < 0.001, Spearman rank correlation coefficient: 0.955) and the number of strong‐binding neoantigens (*p* < 0.001, Spearman rank correlation coefficient: 0.960) were related to the number of SNVs. The number of strong‐binding neoantigens produced by a single mutated gene ranged from zero to nine in all patients, and no specific gene was identified that produced a large number of neoantigens. The mutations that can produce more neoantigens are all individualized. Moreover, none of the top 10 mutation genes produced more than five strong‐binding neoantigens in patients. Additionally, we did not find any neoantigens shared among patients, nor any genes that could produce shared neoantigens. The top 20 mutated genes and mutation patterns in patients with high ITH was shown in Figure 3G.

We then compared the profile of the mutated genes and their generated neoantigens in patients with a worse prognosis with those in patients with a better prognosis. According to a median PFS follow‐up time of 15.0 months, patients who experienced disease progression within this period were classified as having a poor prognosis, whereas those who did not were classified as having a better prognosis. Subsequently, a comparison was made between the mutation profiles of patients in these different prognosis groups. However, no gene was found to exhibit a significant difference in mutation frequency between the two groups. The mutation profiles can be observed in Figure 3H,I. We found that the frequency of IGLV‐3 mutations appeared to be higher in patients with a better prognosis, but the pattern of IGLV‐3 mutations was different among the five patients with a better prognosis. Moreover, IGLV‐3 gene mutation did not produce more than five strong‐binding neoantigens in a single individual. Moreover, there was no significant difference in PFS between the two groups of patients divided by IGLV‐3 mutation or not (Log‐rank *p* = 0.914).

### 3.8 The immune status of patients with different neoantigen levels

In cancer patients, the immune response to neoantigens of tumor cells is one of the core links of antitumor immunity. The overall status of the body's immune system in different patients is a feedback mechanism to different tumor states, and it is also a manifestation of autoimmune disorders and response intensity. Therefore, peripheral blood lymphocyte subsets were examined in the patients with high ITH in this study. Kendell correlation analysis showed that the number of strong‐binding neoantigens produced by SNVs was correlated with the proportion of CD8 + T cells (*p* = 0.015) and NK cells (*p* = 0.013). Kendall τb correlation coefficient was 0.258 and 0.263 separately. Spearman correlation also showed that the number of strong‐binding neoantigens was correlated with the proportion of CD8 + T cells (*p* = 0.012) and NK cells (*p* = 0.011). Meanwhile, the Spearman rank correlation coefficient was 0.378 and 0.385 separately. Similarly, when patients were divided into more‐loss and less‐loss groups according to the loss of strong‐binding neoantigens, the more‐loss group had significantly lower CD8 + T cells (*p* = 0.044) and NK cells (*p* = 0.040) (Figure 1J,K). However, no statistical difference was found for CD4 + T cells (*p* = 0.075). Meanwhile, we did not find any cytokines or complement associated with neoantigens.

### 3.9 Prediction model of tumor immune escape based on the loss of tumor neoantigen

Previous studies found that high ITH is a key factor in tumor immune escape.^3^, ^4^ Significant loss of strong‐binding neoantigens produced by SNVs was observed in MM patients with high ITH. Since the production and binding ability of neoantigens to T cells is an important part of the tumor immune response, we propose that the phenomenon of neoantigen loss is a significant feature of immune escape. A mismatch between the number of neoantigens and ITH suggests that the body is developing an abnormal antitumor‐associated immune response to the tumor. Immune escape can be a parameter that can well evaluate the biological behavior of tumor cells and predict the prognosis of patients. Here, we used a reduction in strong‐binding neoantigens density per unit number of variant allele frequency distribution widths to evaluate the intensity of immune escape in high ITH myeloma. We defined the immune escape index as the difference between the standard strong‐binding neoantigens density of the tumor species and the strong‐binding neoantigens density of an individual: Escape index = sNAL/(sMAD/sMedian)−NAL/(MAD/Median). MAD and Median were calculated as the percentage ratio of median absolute deviation (MAD) and the median of its mutant allele fractions at tumor‐specific mutated loci. The standard strong‐binding neoantigen density of tumor species depends on larger studies in the future, and in this study, we only compared the relative values of the escape index. We constructed Kaplan–Meier survival curves and log‐rank tests to assess the prognosis between the groups. As a result, a higher escape index (*p* = 0.022) was associated with a higher possibility of disease progression (Figure 2D).

## 4 DISCUSSION

Our study preliminarily analyzed the loss of neoantigens in high ITH myeloma. In this study, we searched for high ITH individuals by collecting samples from patients with high tumor burden and whole‐exome sequencing. We presented three important findings. First, the calculation of the MATH score is a good method for determining ITH in MM. Moreover, the value of the MATH score was closely related to the prognosis and treatment response of patients. Compared with other highly mutated tumors, MM has a relatively low mutation burden, but high ITH. Second, in myeloma patients with high ITH, we did not find any loss of heterozygosity in HLA and there is a decrease in strong‐binding neoantigens produced by SNVs that can be recognized by T cells. The decrease of such neoantigens is a key factor for patients with poor prognosis and resistance to first‐line treatment. Third, the loss of neoantigens mainly reflects the antigens with the strong‐binding ability with T cells, which could produce a strong immune response, suggesting that this phenomenon is an important mechanism of tumor immune escape. And the tumor immune escape index can be established by identifying the decrease of neoantigen to determine the prognosis of high ITH myeloma patients.

Previous studies have found that the prognosis of highly heterogeneous tumors caused by low TMB has the worst prognosis.^14^ In our study, we found that there was no correlation between TMB and MATH. Previous studies in the immunotherapy field hold the opinion that tumors with increased TMB present more neoantigens and, thus, are more immunogenic.^40^ However, the prognostic value of TMB for tumors is unclear. Some studies use TMB as a predictor of the efficacy of immunotherapy, but TMB is not suitable for all tumors. Some tumors with similar TMB, express distinct responses to immunotherapy.^12^ And higher TMB does not always effectively express neoantigens that elicit T‐cell immune responses.^41^ TMB was not a good predictor of patient outcomes and cannot predict the response of immunomodulators.^42^, ^43^ MATH score, which describes the ITH of the tumor, is a good biomarker for predicting a tumor's prognosis.^44^

Our findings suggest that the loss of strong‐binding neoantigens produced by SNVs is an important cause of immune escape in high ITH tumors. The activation of the immune response depends on the production of neoantigens, antigen presentation, recognition of neoantigens by T cells, and the immune effect of T cells.^7^, ^8^ In previous studies, the number of immune cell infiltration was used to investigate the strength of antitumor immune response.^45^ However, the number of immune cells cannot indicate that the aggregation of immune cells inevitably has an antitumor effect. An increase in immune cell invasion does not necessarily represent an increased immune response.^45^ By examining neoantigens with high recognition potential and strong binding, as well as the presence of HLA‐LOH, our study illustrates the phenomenon of immune evasion in MM patients with high ITH from a more accurate direction. The sharp increase of heterogeneity in the tumor is accompanied by the sharp decrease of the strong‐binding neoantigens, and the weakened immune response leads to the immune escape of the tumor, which also reveals a parameter for the poor prognosis of the increased heterogeneity. Furthermore, we found that patients with greater neoantigen loss had significant reductions in CD8 + T cells and NK cells. The decrease in the proportion of CD8 + T and NK cells, which are direct effector killer cells directed against the tumor, is also consistent with the expectation that the loss of neoantigens leads to diminished immune effects. Although the immune effect of NK cells is not dependent on MHC, about 90% of the NK cells in the peripheral blood are CD56dim NK cells, which have a strong tumor‐killing effect. Moreover, NK cells are innate lymphoid cells that share many phenotypic and functional features with CD8+ T lymphocytes.^46^ Because of the complex interaction between immune escape and immune response, the relationship between NK cells and neoantigens remains to be studied, but the significant reduction in NK cells is also a reflection of the reduced immune effect. We further analyzed the relationship between the number of neoantigens and the total number of B cells (CD19+ T cells) and total number of T cells (CD3+ T cells) in patients. We found that the number of strong‐binding neoantigens in patients was negatively correlated with B cells (*p* = 0.001, Kendall's τb: −0.344), but not with T cells (*p* = 0.785). Given that MM itself is a mature B cell tumor, B cells in the peripheral blood of patients are affected by the feedback and influence of the tumor itself, so we believe that although there are significant differences in B cells between different groups, they cannot well reflect the special immune status of patients. Previous studies have found a negative correlation between the total number of B cells in peripheral blood and the prognosis of MM patients. The number of B cells in peripheral blood was positively correlated with the burden of tumors in MM patients. A negative correlation was found between B cells and the number of antigens in patients in our study, which is consistent with the conclusions of previous studies. For total T cells, however, there was no significant difference between the groups. Given the significant differences in CD8 + T cells, this result is mostly due to the absence of differences in CD4 + T cells. Peripheral CD4 + T cells contain a variety of subsets with different functions. Hence, further exploration may require extra detailed typing. In the future, additional studies are needed.

The prediction of neoantigens is still progressing rapidly. Earlier studies counted neoantigens produced by all mutant forms together or only neoantigens produced by SNVs. However, SNVs can lead to single amino acid changes, while Indels can lead to larger peptide mutations. Although both types of mutations can trigger immune responses, most studies believe that the two neoantigens elicit different immune responses due to differences in their distribution and degree of aggregation. Neoantigens generated by the two modes of mutation should be treated separately. In our study, an updated classification of neoantigens was used to obtain more accurate results. The research on neoantigens is at the forefront and still needs to be further studied. The immune effects of neoantigens generated by SNVs have also been preliminarily explored, but the immune effects and recognition mechanisms of population neoantigens generated by indels are more heterogeneous and complex, and there is still a lack of consensus and evidence‐based medicine evidence. However, in our study, we did not find meaningful conclusions related to neoantigens generated by indels.

The first‐line treatment for MM patients is a combination of proteasome inhibitors, immunomodulators, and hormones,^47^ and the antitumor immune response is an important part of the antitumor effect after drug ingestion in MM. The body's antitumor immune response to tumors depends on the recognition of tumor‐specific neoantigens by T cells.^8^, ^19^ The increase in tumor malignancy is often accompanied by an increase in heterogeneity.^48^ In our cohort, for patients with high ITH, the number of neoantigens remains highly variable. For patients with more neoantigens, even if they do not receive autologous stem cell transplantation due to renal impairment, poor physical condition, and other reasons, they can still obtain better efficacy and longer PFS. Moreover, as these patients have a better response to treatment, they can try maintenance therapy for a longer period.

For MM, the existing staging and prognosis system is incomplete. Although the concept of cytogenetics is added to the R‐ISS stage, the prognosis of patients is still vague. Besides, patients with the same stage have a great difference in prognosis, especially in response to first‐line therapy. In addition, the existing stage and prognostic indicators are difficult to guide the individual treatment plan of patients. MM is a tumor originating from the immune system and highly sensitive to immunomodulators, with high ITH and interindividual heterogeneity.^49^ Our study focused on patients with high ITH and found that the phenomenon of neoantigen loss was closely related to the duration and depth of remission for patients with first‐line therapy. For patients with low neoantigen loss, long‐term remission can be achieved through drug therapy. But for patients with high neoantigen loss, neither conventional therapy nor immune‐based therapy may provide clinical benefit.

Our study also provides a method to assess the strength of tumor immune escape in MM patients. However, in tumor immune escape, neoantigen expression, antigen presentation, and T cell recognition all play crucial roles. Our immune escape index mainly combines neoantigen expression and T‐cell binding capacity but does not incorporate defects in neoantigen presentation. Currently, the defect in antigen presentation of neoantigens mainly results from the HLA‐LOH. In this study, we investigated the presence of HLA‐LOH in the enrolled MM patients, but no HLA‐LOH was found. Therefore, our immune escape index does not include an antigen presentation defect index. Admittedly, our exponential model of immune escape is not comprehensive and reflects only the loss of neoantigens in patients. However, it remains valuable as one of the important causes of immune escape.

Our study attempted to find the rules of ITH and neoantigen changes by comparing specimens before and after patient progression, but we failed. In relapsed and refractory patients, due to previous treatment and drug‐related stress screening of the patient's cell subsets, there are strong polymorphisms in the ITH of patients. There are also great variations and differences in the changes of neoantigen. The cell biological behavior of relapsed and refractory myeloma is more complex and requires more research in the future.

Limitations of the present study are as follows: First, the sample size of our study was relatively small. As the level of ITH cannot be determined before sequencing, and our study focused on patients with high ITH, so we mainly focused on patients with significant clinical symptoms to improve the detection rate. And for MM, autologous hematopoietic stem cell transplantation is an important factor affecting the treatment and prognosis of patients. Therefore, to better investigate the response to first‐line treatment, we only included patients who did not receive hematopoietic stem cell transplantation, which further limited our sample size. Second, our conclusions lack a queue from other centers for verification. The calculation of neoantigens is based on the original data of individualized next‐generation sequencing. Due to patient privacy protection and the large and difficult access of raw sequencing FASTQ files, validation of neoantigens from external datasets is difficult. Moreover, the mixed inclusion criteria and the imperfect clinical information of the external dataset further increase the difficulty of verification, only MATH scores were validated. Third, currently, tumor neoantigen discovery mainly relies on computer prediction after conducting WES or analyzing protein data. Both methods have their own advantages and disadvantages. However, the current mainstream method primarily relies on WES data. In our study, we also relied on WES data. However, it is worth noting that recent studies have started incorporating RNA‐seq data to refine and filter the neoantigens predicted by WES data, aiming to achieve more accurate conclusions. In our study, we did not perform RNA‐seq assays to filter the neoantigens identified by WES. This is definitely a limitation of our study. However, it is important to note that the main goal of our study was to determine the total number of neoantigens, and the incorporation of RNA‐seq data into neoantigen identification would act as a filtering process, which would have only a minor impact on the final results. Although the lack of RNA‐seq data filtering may have had some effect on the final number of neoantigens, the effect was relatively small. Moreover, many studies still believe that relying solely on WES data also has high accuracy. Our study focused on the overall number of neoantigens rather than identifying individual peptide chains, and therefore, the study still has good confidence. Fourth, since this study is retrospective, there may be more divergence than in a prospective study. However, due to the unpredictability of ITH before next‐generation sequencing, it is difficult to conduct prospective studies to identify highly heterogeneous myeloma patients.

## 5 CONCLUSION

Previous studies confirmed the relevance of tumor neoantigens in the recognition of cancer cells by the immune system. Our study focused on high ITH MM patients with poor prognoses. We found that in myeloma patients with high ITH, there is a decrease in neoantigens that can be recognized by the human immune system. And the loss of neoantigens is mainly reflected in the part of neoantigens that have strong‐binding ability and recognition potential with T cells. The decrease of neoantigens is a key factor for patients with poor prognosis and insensitivity to standard first‐line therapy. Since HLA‐LOH was not found in MM patients with high ITH, we believe that the loss of strong‐binding neoantigens is an important mechanism of immune escape in high ITH MM. Furthermore, we found that patients with greater neoantigen loss had significant reductions in CD8 + T cells and NK cells. A prediction model based on the loss of neoantigens was also built to evaluate the strength of immune escape. The loss of neoantigens based on the gene expression profile may be feasible for deciding the clinical treatment of MM patients in future research.

## AUTHOR CONTRIBUTIONS

**Yue Wang:** Conceptualization (lead); data curation (lead); formal analysis (lead); funding acquisition (equal); investigation (lead); methodology (lead); project administration (lead); resources (lead); software (lead); writing – original draft (lead). **Jiadai Xu:** Data curation (equal); investigation (equal); resources (equal). **Tianwei Lan:** Data curation (equal); formal analysis (equal); investigation (equal). **Chi Zhou:** Data curation (equal); formal analysis (equal); visualization (equal). **Peng Liu:** Conceptualization (equal); data curation (equal); funding acquisition (lead); supervision (equal); validation (equal); writing – review and editing (lead).

## FUNDING INFORMATION

Science and Technology Innovation Action Plan of Shanghai (21YF1406300), National Natural Science Foundation of China (81570123) National Key New Drug Creation Special Programs (2017ZX09304‐021), and Academic Pacesetters Program of Shanghai Healthcare System (2017BR033).

## CONFLICT OF INTEREST STATEMENT

None declared.

## ETHICS STATEMENT

The study was approved by the Ethics Committee of Fudan University Zhong Hospital and complied with the principles of the Helsinki Accord.

## CONSENT TO PARTICIPATE

This was a retrospective study and patient consent was therefore deemed unnecessary. However, bone marrow samples were collected from all subjects, with patient consent, and stored in the tissue bank at Fudan University Zhongshan Hospital.

## CONSENT FOR PUBLICATION

The manuscript is approved by all authors for publication.

## Supporting information


Figures S1–S4

Table S1

Data S1

## Data Availability

Somatic events are available in the supplementary materials. The raw FASTQ data are available and free from corresponding authors upon reasonable request.
