# Supplementary material for: The loss of neoantigens is an important reason for immune escape in multiple myeloma patients with high intratumor heterogeneity
Source: Cancer Med. 2023 Nov 15;12(24):21651–65. doi: 10.1002/cam4.6721 (PMC10757111; doi:10.1002/cam4.6721)
Supplement: Supplementary file 1 — Figures S1–S4 [file CAM4-12-21651-s001.zip › Figure captions.docx]

Supplementary Figure 1. Baseline gene mutation characteristics in the training set.

Supplementary Figure 2. Baseline gene mutation characteristics in the validation set.

Supplementary Figure 3. LASSO regression to identify parameters which related to patients' PFS in validation set.

Supplementary Figure 4. Tumor mutation burden in MM and 33 types of tumor in TCGA database.
