# Supplementary figures and images for: The loss of neoantigens is an important reason for immune escape in multiple myeloma patients with high intratumor heterogeneity
Source: Cancer Med. 2023 Nov 15;12(24):21651–65. doi: 10.1002/cam4.6721 (PMC10757111; doi:10.1002/cam4.6721)

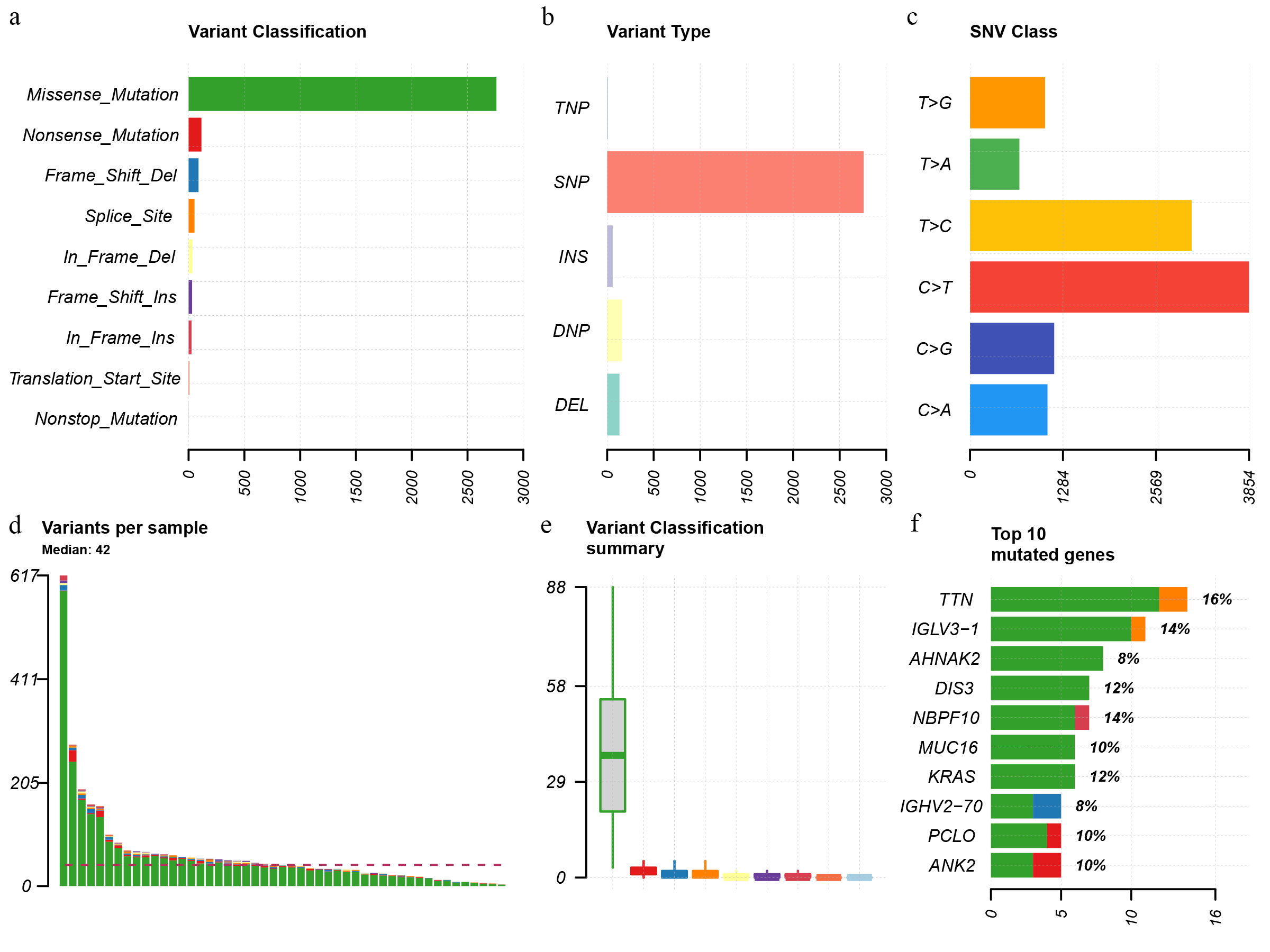

Supplement: Supplementary file 1 — Figures S1–S4 [file CAM4-12-21651-s001.zip › cam46721-sup-0001-FigureS1.tiff]

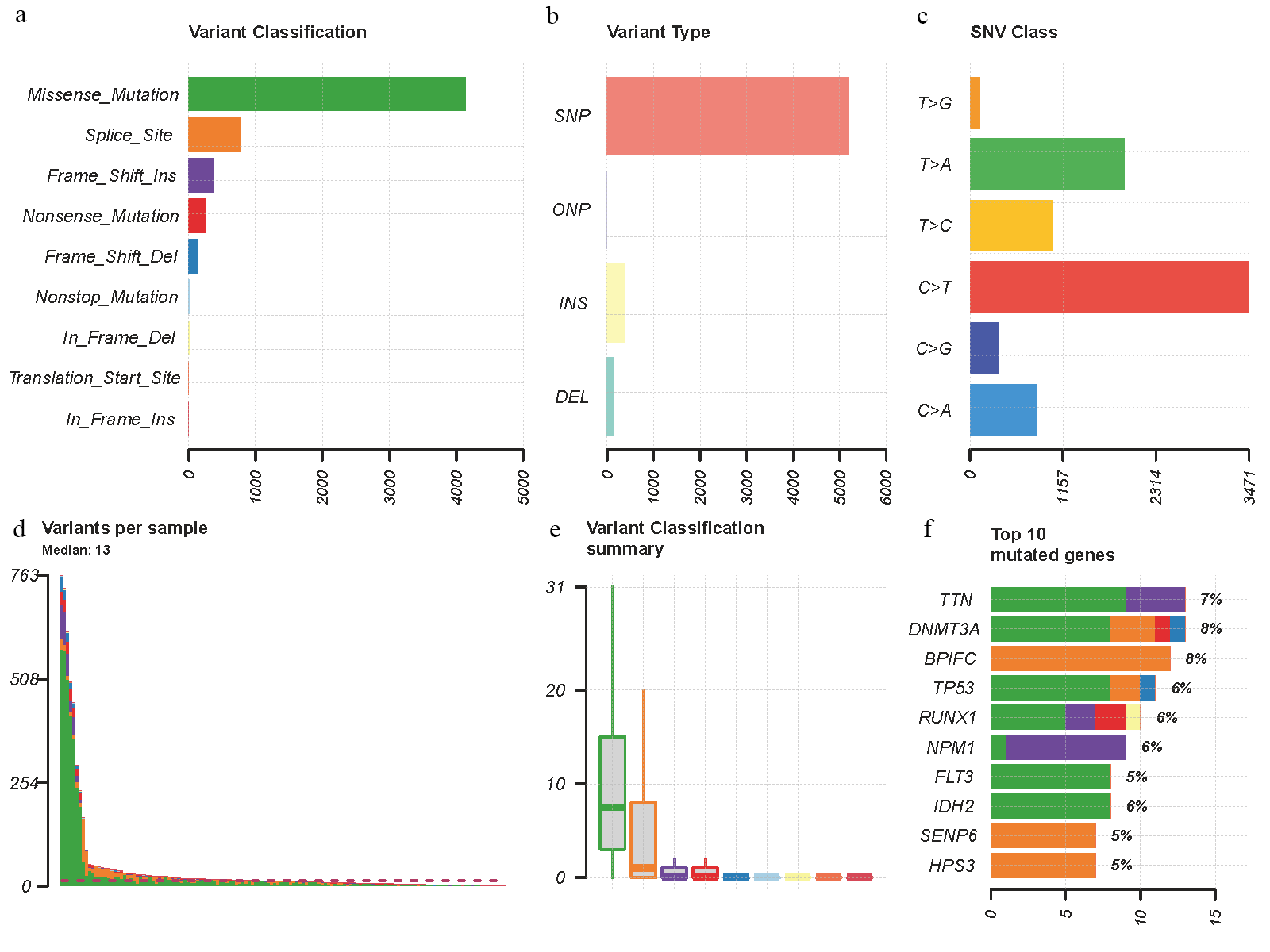

Supplement: Supplementary file 1 — Figures S1–S4 [file CAM4-12-21651-s001.zip › cam46721-sup-0002-FigureS2.tiff]

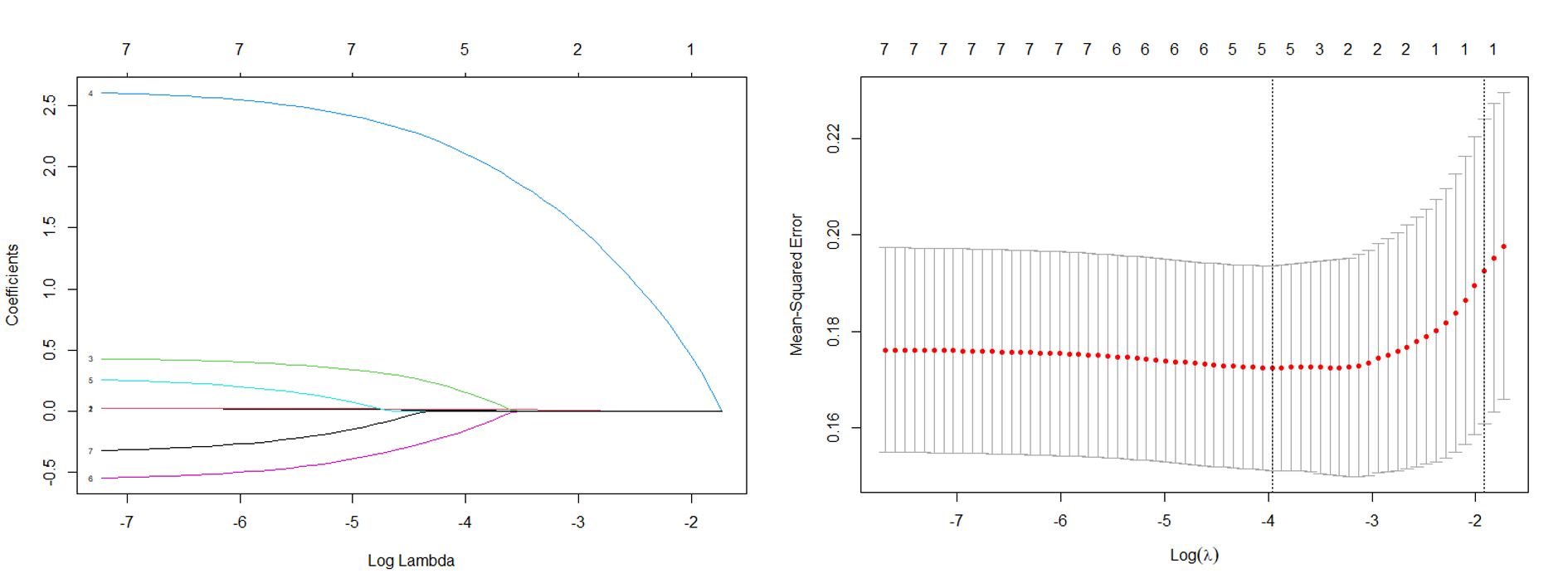

Supplement: Supplementary file 1 — Figures S1–S4 [file CAM4-12-21651-s001.zip › cam46721-sup-0003-FigureS3.tiff]

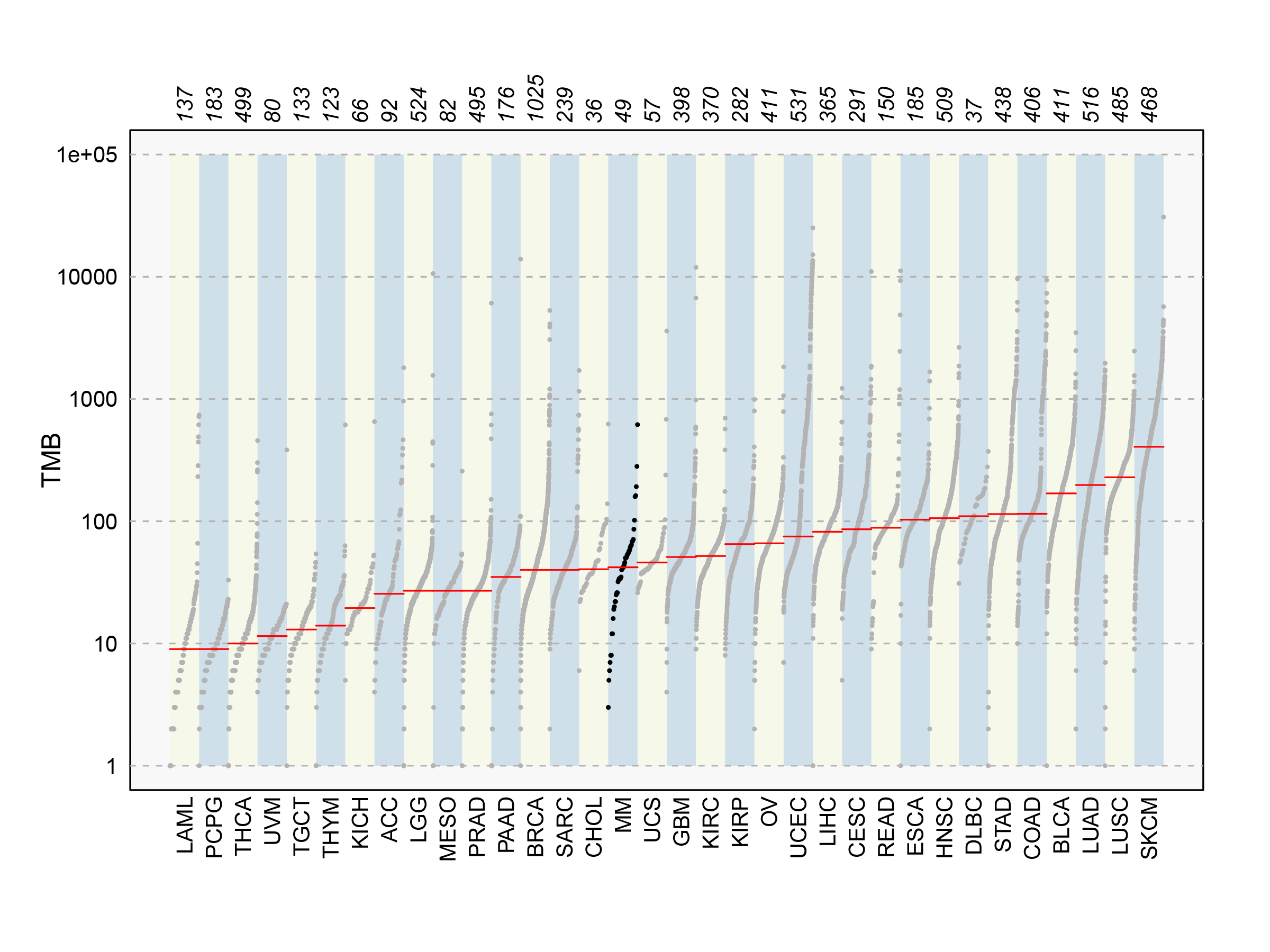

Supplement: Supplementary file 1 — Figures S1–S4 [file CAM4-12-21651-s001.zip › cam46721-sup-0004-FigureS4.tiff]
