# Supplementary material for: The loss of neoantigens is an important reason for immune escape in multiple myeloma patients with high intratumor heterogeneity
Source: Cancer Med. 2023 Nov 15;12(24):21651–65. doi: 10.1002/cam4.6721 (PMC10757111; doi:10.1002/cam4.6721)
Supplement: Supplementary file 2 — Table S1 [file CAM4-12-21651-s003.docx]

**Supplementary Table 1. Baseline characteristics of patients in the validation cohort.**

| Variable | MM |
| --- | --- |
| Patinets(No) | 201 |
| Gender(No,%) |  |
| Male | 128(63.7) |
| Female | 67(33.3) |
| NA | 6(3.0) |
| Age(No,%) |  |
| <60 | 72(35.8) |
| ≥60 | 88(43.8) |
| NA | 41(20.4) |
| Heavychain(No,%) |  |
| IgG | 93(46.3) |
| IgA | 31(15.4) |
| IgM | 1(0.5) |
| FLC | 7(3.5) |
| NA | 68(33.8) |
| Lightchain(No,%) |  |
| kappa | 92(45.8) |
| lamda | 47(23.4) |
| Biphenotypic | 1(0.5) |
| None | 3(1.5) |
| NA | 58(28.9) |
| FISH high-risk(No,%) |  |
| Detected | 50(24.9) |
| Not detected | 151(75.1) |
| Progress(No,%) | 35(17.4) |
| Dead(No,%) | 41(20.4) |
